# Association between obesity and high blood pressure among Lithuanian adolescents: a cross-sectional study

**DOI:** 10.1186/s13052-014-0102-6

**Published:** 2014-12-10

**Authors:** Virginija Dulskiene, Renata Kuciene, Jurate Medzioniene, Rimantas Benetis

**Affiliations:** Institute of Cardiology, Medical Academy, Lithuanian University of Health Sciences, Sukileliu ave. 17, LT-50009 Kaunas, Lithuania

**Keywords:** Blood pressure, Prehypertension, Hypertension, Overweight, Obesity, Abdominal obesity, Adolescents

## Abstract

**Background:**

Most epidemiological studies have shown that the prevalence of high blood pressure (BP) has significantly increased among children and adolescents in various countries of the world.

The aim of this study was to examine the associations between overweight, obesity, abdominal obesity and prehypertension and hypertension among Lithuanian adolescents aged 12–15 years.

**Methods:**

The subjects with increased BP (≥90th percentile) were screened on two separate occasions. Data on the body mass index (BMI), waist circumference (WC), and BP were analysed in 7,457 adolescents aged 12–15 years. Adjusted odds ratios (aORs) with 95% confidence intervals (CI) for the associations were estimated using multivariate logistic regression models.

**Results:**

After two screenings, the study participants were categorised as prehypertensive (12.8%), hypertensive (22.2%), and normotensive (65%). The overall prevalence of overweight, obesity, and abdominal obesity (if WC was in the ≥75th percentile) were 12.1%, 2.4%, and 9%, respectively. After adjusting for age and sex, significant associations were found between overweight and obesity and high BP, namely, prehypertension (overweight: aOR = 2.62; 95% CI 2.13–3.23; obesity: aOR = 4.81; 95% CI 3.08–7.52) and hypertension (overweight: aOR = 3.56; 95% CI 3.02–4.19; obesity: aOR = 6.64; 95% CI 4.65–9.49). Prehypertension was found to be significantly associated with WC in the 75th– < 90th percentiles (aOR = 3.16; 95% CI 2.43–4.10) and WC in the ≥90th percentile (aOR = 4.08; 95% CI 2.35–7.10). For hypertension, significant associations were detected with WC in the 75th– < 90th percentiles (aOR = 3.92; 95% CI 3.18–4.82) and WC in the ≥90th percentile (aOR = 7.41; 95% CI 4.97–11.05).

**Conclusions:**

Overweight, obesity, and abdominal obesity were associated with prehypertension and hypertension.

## Background

Overweight and obesity are serious and growing global public health problems among children and adolescents [1]. Childhood and adolescent obesity is associated with many cardiovascular risk factors: high blood pressure (BP), dyslipidaemia, abnormalities in endothelial function, and hyperinsulinaemia [2]. Abdominal obesity is also related to cardiovascular and metabolic risk factors [3]. Twenty-five longitudinal studies included in the systematic review have reported that overweight and obese youth (children and adolescents) were at an increased risk of becoming overweight or obese in adulthood [4]. It has been shown that childhood overweight and obesity are related to adverse levels of cardiovascular disease risk factors in adulthood [5]. Childhood BP is a significant predictor of adult BP [6]. It is well known that high BP in adulthood increases the risk of cardiovascular disease (such as ischemic heart disease, stroke, or hypertensive heart disease) and is the leading cause of morbidity and mortality worldwide [7]. It is therefore essential to begin to identify the risk factors associated with the development of cardiovascular diseases and other chronic non-communicable diseases, and then make every effort to prevent and control these factors in childhood and adolescence.

Epidemiological studies have reported that the prevalence of high BP has significantly increased among children and adolescents over the recent years [8-12]. Studies have shown that obesity was significantly associated with hypertension [9,13-15] or high BP (≥90th percentile) [16,17] among children and adolescents. Several studies that examined the relationships between overweight, obesity, and prehypertension [9,12,14] have yielded different findings. There has yet been little scientific evidence supporting the associations between abdominal obesity and prehypertension and hypertension in children and adolescents.

In Lithuania, the prevalence of hypertension in the adult population remains high and increases with age [18]. According to the data of the Health Statistics of Lithuania, cardiovascular diseases are the most common cause of death, and their prevalence is among the highest in Europe [19]. Moreover, statistical data show that the prevalence of chronic diseases and various health disorders among children and adolescents has been increasing during the last years [19]. A study conducted in Lithuania has indicated a significant prevalence of elevated BP in children aged 3–7 years (21.4%) [20]. Although the relationships between overweight and increased BP have been established among preschoolers [20], the associations between overweight and obesity with prehypertension and hypertension have not yet been studied in adolescents aged 12–15 years. No epidemiological study in Lithuania has investigated the associations of abdominal obesity as measured by waist circumference (WC) with prehypertension and hypertension among adolescents.

The aim of this study was to evaluate the associations of overweight, obesity, and abdominal obesity with prehypertension and hypertension among adolescents living in Lithuania.

## Materials and methods

### Study population

This cross-sectional study included 12- to 15-year-old adolescents who at the time of the examination (from November 2010 to April 2012) attended gymnasiums or secondary schools in Kaunas city and Kaunas district – the second largest city and district by population size in Lithuania. All the invited schools (n = 81) accepted the invitation to participate in the research project. The schoolchildren who had endocrine diseases, diabetes mellitus, kidney diseases, cardiovascular diseases, or congenital heart defects were excluded from the data analysis. Data on clinically verified health disorders were collected from the subjects’ medical records (Form No. 027-1/a). All medical data were reviewed, and both BP and anthropometric measurements were taken at the participants’ schools by the same team of trained study personnel (physicians and research assistants).

Of 7,638 subjects who participated and were examined in the study, 152 were excluded due to the presence of any of the above-mentioned diseases. Thus, a total of 7,486 participants (3,510 boys and 3,976 girls) met the inclusion criteria. Among these, 29 participants were excluded from the statistical analysis due to missing data on weight, height, or WC.

A written informed consent was obtained from each participant’s parent or guardian. The study was approved by Kaunas Regional Ethics Committee for Biomedical Research at the Lithuanian University of Health Sciences (protocol No. BE-2-69).

### Blood pressure measurements

Blood pressure was measured in the morning hours (8:30 am to 11:30 am) by the physician who was not wearing a white coat. The subjects were advised to avoid tea, coffee, energy drinks, and physical exercises in the morning of the examination day until the measurements were taken. Before the BP measurement, the participants were asked to sit still for ten minutes. BP was measured three times with a 5-minute rest interval between the measurements, with the participant being in a sitting position; BP was measured using an automatic BP monitor (OMRON M6; OMRON HEALTHCARE CO., LTD, Kyoto, Japan) with the appropriate cuff size (17–22 cm; 22–32 cm; 32–42 cm). The average of three BP measurements was calculated. All subjects with increased BP (BP was in the ≥90th percentile; n = 2,597) during the first screening underwent a second evaluation of BP measurements within a period of 2–3 weeks. If BP was ≥90th percentile during both visits, the final BP status was based on the highest average BP values observed during the first or the second screenings.

Classifications and definitions of BP levels were defined according to “The Fourth Report on the Diagnosis, Evaluation, and Treatment of High Blood Pressure in Children and Adolescents” (National High Blood Pressure Education Program (NHBPEP) Working Group on High Blood Pressure in Children and Adolescents) [21] published in 2004. According to BP charts for age, sex, and height*,* normal BP was defined as systolic blood pressure (SBP) and diastolic blood pressure (DBP) <90th percentile; prehypertension was defined as average SBP or DBP levels between the ≥90th percentile and the <95th percentile; and hypertension was defined as average SBP or DBP readings ≥95th percentile.

### Anthropometric measurements

Height and weight of the participants (wearing only light clothing and barefooted) were measured using a portable stadiometer and a balance beam scale (SECA measuring equipment). Height was measured to the nearest 0.1 cm and weight was measured to the nearest 0.1 kg. The body mass index (BMI) was calculated as weight in kilograms divided by the square of height in meters. Waist circumference (WC) was measured with a flexible measuring tape (SECA) at a level midway between the lower rib margin and the iliac crest. WC was measured to the nearest 0.5 cm.

According to the age and sex specific cut-off points of BMI proposed by the International Obesity Task Force [22], the participants were grouped into three categories of BMI: normal-weight, overweight, and obese. The participants were divided into three categories on the basis of their WC: <75th percentile (normal waist value), 75th– < 90th percentile (moderate), and ≥90th percentile (high), using the cut-off values of WC percentiles as defined in the criteria of the Third National Health and Nutrition Examination Survey (NHANES III) [23]. Abdominal obesity among adolescents was defined at moderate and high WC percentile categories (in the 75th– < 90th percentile and ≥90th percentile).

### Statistical analysis

Categorical variables were expressed as numbers (n) and percentages (%), and were compared using the chi-square (*χ*^2^) test. Tests for trend were analysed with the Cochran-Armitage test for trend. The normality of the distribution of continuous variables was tested by the Kolmogorov-Smirnov test. Means and standard deviations (SD) were presented for normally distributed continuous variables, and medians and interquartile ranges (25th–75th percentiles) - for non-normally distributed continuous variables. Comparisons between groups were performed by applying the *t*-test and ANOVA (parametric tests for normally distributed continuous variables), the Mann–Whitney test, and the Kruskal-Wallis test (non-parametric tests for non-normally distributed continuous variables). Univariate and multivariate logistic regression analyses were conducted separately for boys and girls, and for both sexes combined to evaluate the associations between the analysed risk factors such as BMI categories (overweight and obesity), moderate and high WC percentile categories (WC in the 75th– < 90th percentile and WC in the ≥90th percentile) and prehypertension/hypertension. Crude odds ratios (OR) and adjusted odds ratios (aOR) along with 95% confidence intervals (CI) were calculated. In multivariate analyses conducted for boys and girls separately, ORs were adjusted for age*.* In the multivariate analysis for both sexes combined, ORs were adjusted for age and sex.

Statistical analyses were performed using the statistical software package SPSS version 20 for Windows. P values < 0.05 were considered to be statistically significant.

## Results

The final study sample consisted of 7,457 subjects (of whom 46.9% (n = 3,494) were boys, and 53.1% (n = 3,963) were girls) aged from 12 to 15 years. The median values of demographic (age), anthropometric (weight, height, BMI, and WC), and BP (SBP and DBP) data for the whole sample and for each sex separately are presented in Table 1. Boys were significantly heavier and taller, and had a higher median BMI and WC. They had a significantly higher median SBP and a significantly lower median DBP, compared to girls. The median age was similar for boys and girls.

**Table 1:** **Demographic, anthropometric, and BP characteristics of the study participants by sex**

**Variables**	**Total**	**Boys**	**Girls**	**p** ^*^
	**(n = 7,457)**	**(n = 3,494)**	**(n = 3,963)**	
Age (years)	13.0 (12.0–14.0)	13.0 (12.0–14.0)	13.0 (12.0–14.0)	0.924
Weight (kg)	52.0 (45.0–60.0)	54.4 (45.0–64.0)	51.0 (45.0–58.0)	<0.001
Height (cm)	164.5 (158.0–171.0)	167.0 (158.0–175.0)	163.0 (158.0–168.0)	<0.001
BMI (kg/m^2^)	19.1 (17.4–21.2)	19.2 (17.5–21.3)	19.0 (17.3–21.1)	<0.001
WC (cm)	66.0 (62.0–71.0)	68.0 (64.0–73.0)	64.0 (60.5–68.0)	<0.001
SBP (mmHg)	115.3 (108.3–126.7)	117.7 (110.7–133.3)	114.0 (107.3–120.7)	<0.001
DBP (mmHg)	65.0 (60.3–70.3)	64.3 (59.7–70.0)	65.3 (60.7–70.7)	<0.001

BP – blood pressure, BMI – body mass index, WC – waist circumference, SBP – systolic blood pressure, DBP – diastolic blood pressure.

Values are presented as median (25th–75th percentiles).

^*^Boys versus girls.

Table 2 presents the basic characteristics of the participants according to BP level. Overall, the prevalence rates of prehypertension and hypertension were 12.8% (15.0% for boys and 10.8% for girls) and 22.2% (29.1% for boys and 16.1% for girls), respectively. Girls were more often normotensive than boys (73.1% and 55.9%, respectively). Older participants (aged 14–15 years) were significantly more likely to have prehypertension and hypertension than younger participants (aged 12–13 years) did (43.4% versus 27.3%). The overall prevalence of overweight and obesity was 12.1% (14.3% for boys and 10.2% for girls) and 2.4% (2.7% for boys and 2.2% for girls), respectively. The prevalence of WC in the 75th– < 90th percentile and WC ≥90th percentile was, respectively, 7.1% and 1.9% of the entire group of the study participants. Overall, 7.3% of the participants were overweight/obese as well as abdominally obese. Among 670 subjects with abdominal obesity (WC ≥75^th^ percentile), there were 19.0%, 57.1% and 23.9% participants who were normal-weight, overweight, and obese, respectively. Prehypertensive and hypertensive participants had significantly higher median values for weight, height, BMI, WC, SBP, and mean value for DBP, compared to normotensive participants. The median weight was higher among hypertensive adolescents than among prehypertensive participants, but no significant difference between these groups was found (Table 2).

**Table 2:** **Characteristics of the study participants according to BP level**

**Variables**	**Normotensive**		**Prehypertensive**		**Hypertensive**		***p***
	**(n = 4,847)**		**(n = 955)**		**(n = 1,655)**		
	**n**	**%**	**n**	**%**	**n**	**%**	
Sex:							
Boys	1,952	55.9	524	15.0	1,018	29.1	<0.001* ^
Girls	2,895	73.1	431	10.8	637	16.1	
Age (years):							
12–13	2,823	72.7	322	8.3	737	19.0	<0.001* ^
14–15	2,024	56.6	633	17.7	918	25.7	
BMI categories:							
Normal weight	4,409	69.2	759	11.9	1,204	18.9	<0.001* ^
Overweight	384	42.4	159	17.6	362	40.0	
Obesity	54	30.0	37	20.6	89	49.4	
WC percentile categories:							
<75th	4,601	67.8	831	12.2	1,355	20.0	<0.001* ^
75th– < 90th	205	38.5	102	19.2	225	42.3	
≥90th	41	29.7	22	15.9	75	54.4	
Age (years)	13.0 (12.0–14.0)		14 (13.0–15.0)^a^		14.0 (13.0–15.0)^a,b^		<0.001^#^
Weight (kg)	50.0 (42.0–56.0)		58.0 (52.0–66.0)^a^		59.0 (52.0–68.0)^a^		<0.001^#^
Height (cm)	162.0 (156.0–168.0)		170.0 (164.0–176.0)^a^		168.0 (161.5–175)^a,b^		<0.001^#^
BMI (kg/m^2^)	18.5 (16.9–20.2)		20.1 (18.4–22.1)^a^		20.7 (18.9–22.9)^a,b^		<0.001^#^
WC (cm)	64.0 (61.0–69.0)		68.0 (64.0–73.0)^a^		70.0 (65.0–75.0)^a,b^		<0.001^#^
SBP (mmHg)	111.0 (105.3–115.0)		124.3 (122.0–127.3)^a^		138.0 (132.7–144.3)^a,b^		<0.001^#^
DBP (mmHg)	63.1 ± 6.4		67.1 ± 6.9^a^		71.7 ± 8.1^a,b^		<0.001^#^

BP – blood pressure, BMI – body mass index, WC – waist circumference, SBP – systolic blood pressure, DBP – diastolic blood pressure, SD – standard deviation.

Values are presented as mean ± SD or median (25th–75th percentiles).

^a^Significantly different (p < 0.05) from normotensive participants.

^b^Significantly different (p < 0.05) from prehypertensive participants.

*Significant difference using chi-square (*χ*2) test.

^Significant difference using Cochran-Armitage trend test.

^#^Significant difference between three groups.

The median values of BP and anthropometric variables (including weight, BMI, and WC) increased with increasing BMI (Table 3) and WC (Table 4).

**Table 3:** **Characteristics of the study participants according to BMI categories**

	**Normal weight**	**Overweight**	**Obesity**	p*
***Boys***	**(n = 2,901)**	**(n = 499)**	**(n = 94)**	
Age (years)	13.0 (12.0–14.0)	13.0 (12.0–14.0)^a^	13.0 (12.0–14.0)	<0.001
Weight (kg)	52.0 (43.8–60.0)	68.0 (60.0–75.0)^a^	83.5 (72.5–90.4)^a, b^	<0.001
Height (cm)	167.0 (158.0–175.0)	168.0 (160.0–176.0)^a^	167.8 (160.0–175.0)	0.037
BMI (kg/m^2^)	18.61 ± 2.00	23.90 ± 1.57^a^	29.60 ± 2.91^a,b^	<0.001
WC (cm)	67.0 (63.0–71.0)	77.0 (72.0–82.0)^a^	89.0 (82.0–96.0)^a, b^	<0.001
SBP (mmHg)	116.3 (109.3–130.7)	128.7 (116.0–139.3)^a^	131.2 (118.6–140.8)^a^	<0.001
DBP (mmHg)	64.0 (59.3–69.3)	66.0 (61.3–71.7)^a^	69.0 (63.3–75.0)^a, b^	<0.001
***Girls***	**(n = 3,471)**	**(n = 406)**	**(n = 86)**	
Age (years)	13.0 (12.0–14.0)	13.0 (12.0–14.0)	13.0 (12.0–14.0)	0.06
Weight (kg)	50.0 (44.0–55.0)	66.0 (60.0–70.0)^a^	80.0 (75.8–89.0)^a, b^	<0.001
Height (cm)	163.0 (158.0–168.0)	164.0 (159.0–169.0)	163.0 (160.0–168.0)	0.284
BMI (kg/m^2^)	18.6 (17.0–20.1)	24.4 (23.5–25.5)^a^	29.8 (28.6–32.2)^a, b^	<0.001
WC (cm)	63.0 (60.0–67.0)	74.0 (70.0–79.0)^a^	87.0 (81.0–90.0)^a, b^	<0.001
SBP (mmHg)	113.0 (106.7–119.0)	119.0 (112.3–130.1)^a^	125.5 (117.0–135.0)^a, b^	<0.001
DBP (mmHg)	65.0 (60.3–70.3)	67.8 (63.0–73.7)^a^	70.0 (64.6–75.5)^a, b^	<0.001
***All participants***	**(n = 6,372)**	**(n = 905)**	**(n = 180)**	
Age (years)	13.0 (12.0–14.0)	13.0 (12.0–14.0)^a^	13.0 (12.0–14.0)^a^	<0.001
Weight (kg)	50.0 (44.0–57.0)	67.0 (60.0–73.0)^a^	82.0 (74.0–89.0)^a, b^	<0.001
Height (cm)	164.0 (158.0–171.0)	165.0 (159.0–172.0)^a^	165.0 (160.0–172.0)	<0.001
BMI (kg/m^2^)	18.6 (17.1–20.1)	24.1 (23.0–25.3)^a^	29.3 (28.1–31.6)^a, b^	<0.001
WC (cm)	65.0 (61.0–69.0)	76.0 (71.0–80.0)^a^	87.0 (81.3–93.8)^a, b^	<0.001
SBP (mmHg)	114.7 (107.7–124.0)	123.7 (114.7–135.3)^a^	128.5 (117.8–138.6)^a, b^	<0.001
DBP (mmHg)	64.7 (60.0–70.0)	67.0 (62.3–72.5)^a^	69.7 (64.0–75.3)^a, b^	<0.001

BMI – body mass index, SD – standard deviation, WC – waist circumference, SBP – systolic blood pressure, DBP – diastolic blood pressure.

Values are presented as mean ± SD or median (25th–75th percentiles).

^a^Significantly different (p < 0.05) from normal weight participants.

^b^Significantly different (p < 0.05) from overweight participants.

*Differences between three groups.

**Table 4:** **Characteristics of the study participants according to WC categories**

	**Normal WC**	**Moderate abdominal obesity**	**High abdominal obesity**	p*
***Boys***	**(n = 3,100)**	**(n = 318)**	**(n = 76)**	
Age (years)	13.0 (12.0–14.0)	13.0 (12.0–14.0)^a^	13.0 (12.0–14.0)^a^	<0.001
Weight (kg)	53.0 (44.3–60.5)	68.8 (61.0–78.0)^a^	80.0 (70.3–91.8)^a, b^	<0.001
Height (cm)	166.0 (157.0–175.0)	170.0 (161.0–177.0)^a^	171.0 (163.0–178.8)^a^	<0.001
BMI (kg/m^2^)	18.8 (17.3–20.5)	23.7 (22.2–25.8)^a^	27.9 (25.3–30.5)^a, b^	<0.001
WC (cm)	67.0 (63.0–71.0)	81.0 (78.5–84.1)^a^	94.0 (90.0–97.8)^a, b^	<0.001
SBP (mmHg)	116.7 (109.7–131.0)	130.3 (117.0–139.8)^a^	133.0 (123.7–140.6)^a^	<0.001
DBP (mmHg)	64.0 (59.3–69.3)	66.8 (62.3–72.7)^a^	70.8 (64.8–75.5)^a, b^	<0.001
***Girls***	**(n = 3,687)**	**(n = 214)**	**(n = 62)**	
Age (years)	13.0 (12.0–14.0)	13.0 (12.0–14.0)^a^	12.5 (12.0–13.0)^a, b^	<0.001
Weight (kg)	50.0 (44.0–56.0)	67.0 (59.8–75.0)^a^	77.3 (69.8–87.3)^a, b^	<0.001
Height (cm)	163.0 (158.0–168.0)	165.5 (160.0–170.0)^a^	163.0 (158.9–168.0)^b^	<0.001
BMI (kg/m^2^)	18.8 (17.2–20.5)	24.5 (22.7–26.5)^a^	29.1 (26.7–32.4)^a, b^	<0.001
WC (cm)	63.5 (60.0–67.0)	80.0 (77.0–83.0)^a^	88.3 (86.8–93.0)^a, b^	<0.001
SBP (mmHg)	113.3 (107.0–119.7)	119.5 (113.9–129.7)^a^	122.2 (116.7–136.8)^a, b^	<0.001
DBP (mmHg)	65.3 (60.3–70.7)	68.8 (64.0–74.0)^a^	70.0 (63.9–75.6)^a^	<0.001
***All participants***	**(n = 6,787)**	**(n = 532)**	**(n = 138)**	
Age (years)	13.0 (12.0–14.0)	13.0 (12.0–14.0)^a^	13.0 (12.0–14.0)^a, b^	<0.001
Weight (kg)	51.0 (44.0–58.0)	68.0 (60.0–76.0)^a^	78.0 (70.0–88.3)^a, b^	<0.001
Height (cm)	164.0 (158.0–171.0)	168.0 (160.3–174.0)^a^	166.5 (160.0–173.3)^a^	<0.001
BMI (kg/m^2^)	18.8 (17.2–20.6)	24.1 (22.3–26.0)^a^	28.2 (25.7–31.5)^a, b^	<0.001
WC (cm)	65.0 (61.0–69.0)	81.0 (78.0–84.0)^a^	91.0 (87.0–97.0)^a, b^	<0.001
SBP (mmHg)	114.7 (108.0–125.0)	126.0 (115.7–136.3)^a^	131.0 (118.0–139.3)^a, b^	<0.001
DBP (mmHg)	64.7 (60.0–70.0)	68.0 (63.0–73.3)^a^	70.3 (64.3–75.4)^a, b^	<0.001

WC – waist circumference, SD – standard deviation, BMI – body mass index, SBP – systolic blood pressure, DBP – diastolic blood pressure.

Values are presented as median (25th–75th percentiles).

^a^Significantly different (p < 0.05) from participants with WC in the <75th percentile.

^b^Significantly different (p < 0.05) from participants with WC in the 75th– < 90th percentile.

*Differences between three groups.

Univariate analysis revealed that overweight and obesity were significantly associated with prehypertension and hypertension for both sexes separately and for the combined group consisting of both boys and girls (Table 5), compared to normal-weight participants. WC in the 75th– < 90th percentile was significantly associated with prehypertension and hypertension for boys and girls separately, and for both sexes combined, compared to participants with WC <75th percentile. WC ≥90th percentile was significantly associated with both types of elevated BP for the group of boys and for the combined group of both sexes. P values were below 0.001 for all the above-mentioned associations. However, WC ≥90th percentile was not significantly associated with prehypertension among girls (p = 0.149).

**Table 5:** **Associations between categories of BMI, WC and prehypertension and hypertension (univariate and multivariate analyses)**

**Variables**	**Prehypertension**		**Hypertension**	
	**OR(95% CI)**	**aOR** ^**1**^ **(95% CI)**	**OR(95% CI)**	**aOR** ^**1**^ **(95% CI)**
***Boys***				
BMI categories:				
Normal weight	1.00	1.00	1.00	1.00
Overweight	2.27 (1.72–2.99)	2.85(2.12–3.84)	3.29(2.65–4.08)	3.83(3.06–4.79)
Obesity	3.32(1.78–6.21)	4.46(2.27–8.75)	5.55(3.37–9.12)	6.52(3.91–10.88)
WC percentile categories:				
<75th	1.00	1.00	1.00	1.00
75th– < 90th	2.76(1.98–3.85)	3.80(2.64–5.46)	3.73(2.86–4.87)	4.67(3.53–6.16)
≥90th	4.44(2.13–9.26)	6.10(2.76–13.45)	7.61(4.16–13.89)	9.95(5.37–18.42)
***Girls***				
BMI categories:				
Normal weight	1.00	1.00	1.00	1.00
Overweight	2.43(1.81–3.27)	2.52(1.87–3.40)	3.35(2.64–4.26)	3.40(2.67–4.33)
Obesity	4.71(2.63–8.43)	5.17(2.86–9.32)	6.50(3.98–10.61)	6.67(4.08–10.91)
WC percentile categories:				
<75th	1.00	1.00	1.00	1.00
75th– < 90th	2.49(1.70–3.66)	2.70(1.83–3.99)	3.13(2.28–4.30)	3.23(2.35–4.45)
≥90th	1.85^NS^(0.80–4.28)	2.41(1.03–5.61)	5.28(3.09–9.03)	5.56(3.24–9.53)
	**OR(95% CI)**	**aOR** ^**2**^ **(95% CI)**	**OR(95% CI)**	**aOR** ^**2**^ **(95% CI)**
***All participants***				
BMI categories:				
Normal weight	1.00	1.00	1.00	1.00
Overweight	2.41(1.97–2.94)	2.62(2.13–3.23)	3.45(2.95–4.04)	3.56(3.02–4.19)
Obesity	3.98(2.60–6.09)	4.81(3.08–7.52)	6.04(4.28–8.51)	6.64(4.65–9.49)
WC percentile categories:				
<75th	1.00	1.00	1.00	1.00
75th– < 90th	2.76(2.15–3.53)	3.16(2.43–4.10)	3.73(3.06–4.55)	3.92(3.18–4.82)
≥90th	2.97(1.76–5.01)	4.08(2.35–7.10)	6.21(4.23–9.13)	7.41(4.97–11.05)

BMI – body mass index, WC – waist circumference.

OR – odds ratio; aOR^1^ – adjusted odds ratio for age; aOR^2^ – adjusted odds ratio for age and sex; CI – confidence interval.

All results were significant at p < 0.001, except when noted (NS – not significant).

The overall significance levels of the multiple logistic regression models were p < 0.001.

The associations identified by univariate analysis were examined further by multivariate logistic regression analysis (Table 5). For prehypertension and hypertension, the age-adjusted ORs among obese adolescents were higher (among boys: aOR = 4.46 and aOR = 6.52, respectively, and among girls: aOR = 5.17 and aOR = 6.67, respectively) than among overweight participants (among boys: aOR = 2.85 and aOR = 3.83, respectively, and among girls: aOR = 2.52 and aOR = 3.40, respectively). After adjustment for age, statistically significant elevated aORs were observed for associations between WC in the 75th– < 90th percentile and prehypertension and hypertension in both sexes (among boys: aOR = 3.80 and aOR = 4.67, respectively, and among girls: aOR = 2.70 and aOR = 3.23, respectively). WC ≥90th percentile was also significantly associated with higher odds of both types of elevated BP for both sexes separately: among boys, the age-adjusted ORs ranged from 6.10 (for prehypertension) to 9.95 (for hypertension), while among girls – from 2.41 (for prehypertension) to 5.56 (for hypertension).

Significant interactions were found between sex and age, sex and BMI, and sex and WC separately for both prehypertension and hypertension (all p < 0.001). Being a boy (vs. a girl) and overweight/obese (vs. normal weighted) at the same time would further increase the individual’s odds of being prehypertensive (OR = 3.11; p < 0.001) and hypertensive (OR = 5.03; p < 0.001). The odds of being prehypertensive (OR = 3.88; p < 0.001) and hypertensive (OR = 6.09; p < 0.001) would also be further increased by a combination of male sex (vs. female) and abdominal obesity (vs. normal WC value) (data not shown).

Table 5 presents the results of multivariate analysis for the combined group of both sexes. The associations also remained significant after adjustments for age and sex in the final multivariate models. Overweight was associated with prehypertension (aOR = 2.62) and hypertension (aOR = 3.56). Significant associations with obesity were observed for prehypertension (aOR = 4.81) and hypertension (aOR = 6.64). In the multivariate analysis, after adjustment for age and sex, the participants with WC in the 75th– < 90th and >90th percentiles were significantly more likely to have prehypertension or hypertension (all p values were <0.001) than participants with WC <75th percentile did. According to the final multivariate models, the participants with WC in the 75th– < 90th percentile had higher odds of prehypertension and hypertension (aOR = 3.16 and aOR = 3.92, respectively). WC ≥90th percentile was associated with 4.08-fold higher odds of prehypertension, and by 7.41-fold higher odds of hypertension (both p < 0.001).

## Discussion

To our knowledge, this is the first report in Lithuania investigating the associations between the selected anthropometric measures of overweight, obesity (based on BMI), abdominal obesity (based on WC), and high BP among schoolchildren aged 12–15 years. The multivariate logistic regression analysis (for both sexes combined) of our data showed significant associations between overweight, obesity, abdominal obesity and prehypertension and hypertension among adolescents.

According to our data, the prevalence of prehypertension and hypertension in adolescents was 12.8% and 22.2%, respectively. Studies on prehypertension and hypertension [8,9,11,12,14,24-27] among children and adolescents of various age groups and different sample sizes showed that the prevalence rates vary widely. A study conducted in China reported that the prevalence of prehypertension and hypertension was, accordingly, 15% and 20.2% among children and adolescents aged 5–18 years [12], which is partially similar to the estimated prevalence in the current study. Several studies also indicated a high prevalence of prehypertension and hypertension in Portuguese adolescents (13.3% and 22%, respectively [25] and 12% and 34%, respectively [26]) and in Iranian adolescent girls (13.9% and 19.4%, respectively [27]). Besides, our results that were obtained in the sample of schoolchildren could be partly explained by a high prevalence of hypertension in Lithuanian adult population [18].

The prevalence of overweight and obesity was higher in the current sample (12.1% and 2.4%, respectively), compared to the data from a cross-sectional survey of youth (10–16 years) from 34 participating countries (the Health Behaviour in School-Aged Children Study) conducted in 2001–2002 [28]. Researchers indicated that the prevalence of overweight and obesity among the studied youth in Lithuania were 5.1% and 0.4% [28]. However, the prevalence of overweight and obesity in our study sample was lower than that in most other studies [8,9,11,12,16], but was higher than in several others [14,29] that investigated risk factors associated with high BP among children and adolescents.

The findings of the current study indicating that overweight and obesity among adolescents were significantly associated with prehypertension are consistent with the results of other previously published studies [8,14]. However, some studies presented different results. One study in China found overweight and obesity to be significantly associated with prehypertension in boys, but not in girls [12]. Another study in Canada reported no significant associations between overweight and prehypertension among girls and across both sexes combined, except among boys; however, obesity was associated with prehypertension across all sex groups [9], the latter result being similar to ours. The findings of the present study were also consistent with the findings of the previous studies that established significant associations for hypertension with overweight [13-15,30] and with obesity [9,13-15], although there are some differences among studies in the criteria for defining overweight and obesity, the subjects’ age, and the number of BP measurements. However, one study did not find any significant association between overweight and hypertension among girls in the population of children in rural Canada [9].

Regarding abdominal obesity, the findings of the present study are not easily comparable with other previous studies because there are differences in sample size, the age of the investigated children and adolescents, the methodology, the cut-off criteria for defining abdominal obesity, and potential confounders (e.g., age and sex). There is a problem concerning the definition of abdominal obesity among children and adolescents in research studies of this field.

The prevalence of abdominal obesity (combined 75th– < 90th and ≥90th WC percentiles) in our study (9%) was almost similar to that reported by Souza et al. (WC >75th percentile: 9.3%) [31], and was lower than that reported in other studies – 22.8% of participants with WC ≥75th percentile [16], and 51.7% of participants with WC >75th percentile [32].

The associations between abdominal obesity and high BP among children or adolescents were established by several researchers [12,16,29,33]; however, these studies yielded inconsistent findings. For example, significant associations between WC ≥90th percentile (defining participants with WC <90th percentile as the reference group) and elevated BP were found in the studies in such countries as Egypt [29], China [33], and the United States [34]. In contrast, Guo et al. [12] found no significant association between WC ≥90th percentile and prehypertension in boys and girls separately, when compared to WC <90th percentile. The data of our study and another published study [16] indicated that the subjects with WC in the 75th– < 90th and ≥90th percentiles may have an increased aOR of high BP, compared to those with WC <75th percentile. In the univariate and multivariate analyses of data from our study for both sexes combined, moderate and high WC percentile categories were associated with elevated BP, except for WC ≥90th percentile with prehypertension, at significantly higher ORs than overweight and obesity measured by BMI. These results are partially consistent with the results of univariate analysis in the study by Flores-Huerta et al. [16]. The results of the multivariate analysis in the above-mentioned study [16] indicated that WC ≥90th percentile – but not in the 75th– < 90th percentile – was significantly associated with an elevated BP. However, in the current study, the associations were established separately for prehypertension and hypertension. Our study revealed that in our study sample, WC in the 75th– < 90th and ≥90th percentiles was significantly associated with prehypertension and hypertension for both sexes combined. WC measurement (which has not yet been performed in Lithuania) could be used in clinical practice to identify adolescents who have an increased risk for the development of cardiovascular diseases.

Indeed, high BP can be caused by various risk factors: environmental factors, genetic factors, and interactions between genetic and environmental factors [35]. BP, plasma glucose, and lipids can exert a dose-dependent effect on cardiovascular risk [36]. Scientific studies have also examined the associations of genetic polymorphisms with an increased risk of hypertension and cardiovascular diseases [37-40]. Results from a recent study [37] have shown that calcium/calmodulin-dependent kinase IV (CaMKIV) plays an essential role in BP regulation through the control of the activity of endothelial nitric oxide synthase. Another study has reported that the angiotensinogen AGT 235 T allele constitutes an independent risk factor for resistant hypertension [40]. Additional genetic and epidemiological investigations regarding the risk of cardiovascular diseases could provide more scientific evidence, which is needed for the development of diagnostic, treatment, and prevention strategies.

Our study has a few limitations. The data analysis of the subjects with increased BP (≥90th percentile) was limited to two separate occasions, after which the participants were classified as prehypertensive or hypertensive, relying on BP measurements. Further screening of BP measurements and detailed clinical examinations are needed for the verification of the diagnosis of prehypertension or hypertension. Moreover, according to the Fourth Report, an elevated BP reading obtained with an oscillometric device should be repeated by using auscultation [21]; however, in the present study, BP readings were obtained by an automatic oscillometric monitor, which has been clinically validated [41]. Other limitations of the current study are that pubertal status, biochemical parameters, and socioeconomic factors were not assessed either. This study investigated the population sample of 12–15 year-old adolescents. Further studies are also needed to examine samples of younger children and older adolescents in our country. It is essential to carry out further research in Lithuania because it could reflect the children’s and adolescents’ real state of health, and would also identify other risk factors for elevated BP. Additionally, future research is required to evaluate the importance of genetic polymorphisms in the increased risk of hypertension among Lithuanian children and adolescents.

Despite the above-mentioned limitations, the results of our study emphasized a high prevalence of prehypertension and hypertension among Lithuanian adolescents, and confirmed that overweight, obesity, and abdominal obesity are associated with high BP. Therefore, public health strategies should focus on the prevention of these risk factors of cardiovascular diseases among schoolchildren. The majority of previous studies have suggested that adequate consumption of dairy products [42], fish, fruit and vegetables, a restricted intake of salt (sodium chloride) [43], and reduced consumption of sugar-sweetened beverages [44] can reduce the risk of hypertension and other cardiometabolic disorders. Recent analysis has also shown that higher levels of physical activity decrease the risk of cardiovascular diseases [45]. The promotion of long-lasting behavioural changes related to healthy nutrition, sufficient physical activities, and other healthy lifestyle factors for preventing and controlling overweight, obesity, abdominal obesity, and elevated BP is highly important.

## Conclusions

According to our data, a high prevalence of high BP was observed among 12–15 year-old adolescents living in Lithuania. Overweight, obesity, and abdominal obesity were significantly associated with prehypertension and hypertension among Lithuanian schoolchildren. These findings would be useful in the development of public health programs for reducing risk factors of cardiovascular diseases, and would also be important for the prevention, management, and treatment of high BP among adolescents.

## Abbreviations

aOR: Adjusted odds ratio
BMI: Body mass index
BP: Blood pressure
CI: Confidence interval
DBP: Diastolic blood pressure
OR: Odds ratio
SBP: Systolic blood pressure
SD: Standard deviation
WC: Waist circumference
